# Effect of Kaempferol on the Biological Behavior of Human Colon Cancer via Regulating MMP1, MMP2, and MMP9

**DOI:** 10.1155/2022/2841762

**Published:** 2022-09-13

**Authors:** Xiaoyu Zhang, Jianchun Fan, Fei Guo, Hailu Huang, Jun Xue, Xueliang Wu, Tian Li

**Affiliations:** ^1^Graduate School, Hebei North University, Zhangjiakou 075000, China; ^2^Department of General Surgery, The First Affiliated Hospital of Hebei North University, Zhangjiakou 075000, China; ^3^Department of Pain Clinic, The First Affiliated Hospital of Hebei North University, Zhangjiakou 075000, China; ^4^School of Basic Medicine, Fourth Military Medical University, Xi'an 710032, China

## Abstract

Kaempferol is a kind of flavonoid, which has a significant anticancer effect. MMPs were discovered with the function of cleaving the extracellular matrix. We utilized bioinformatics to analyze the association and bonding mode between the traditional Chinese medicine (TCM) monomer composition (i.e., kaempferol) and the target proteins. The purpose of our research was to verify the effect of kaempferol on the biological behavior of human colon cancer cells HCT116 and HT29 and the expression of matrix metalloproteinase (MMP) 1, 2, and 9 genes. We detected the changes in the biological behavior of colon cancer cells treated with kaempferol by CCK-8, wound healing assay, transwell migration/invasion assay, and flow cytometry. Meanwhile， we detected the expression difference of the target gene by qRT-PCR and western blot. Compared with the two control groups, the cell viability of the kaempferol group decreased, the rate of cell migration and the number of transmembrane cells in the kaempferol group decreased significantly, and the early apoptosis rate increased, the number of cells in the G1 phase increased and in the S phase decreased. The results of qRT-PCR and western blot showed that the expression of target genes MMP1, 2, and 9 in the kaempferol group was lower than that in the two control groups. Kaempferol can significantly inhibit the proliferation, invasion, and migration ability of colon cancer cells; induce their apoptosis; and block the cell cycle. Meanwhile, the expression of MMP1, 2, and 9 genes was downregulated, which verified the results of bioinformatic analysis.

## 1. Introduction

According to the latest data analysis, globally, lung cancer is still the most important cancer (11.55%), while colorectal cancer (10.2%) has become the third largest cancer after lung cancer [1]. The prognosis of colorectal cancer depends on the tumor stage. The 5-year survival rate of stage I patients is 90%, while that of stage IV patients is only 10%. Although 60% of patients can be surgically removed after diagnosis, 20∼25% of patients still have tumor recurrence and metastasis after surgery and chemotherapy, resulting in death [2]. The effort against colorectal cancer has led to the emergence of various treatment options. At present, surgical resection, chemotherapy, radiotherapy, and targeted therapy are adopted in clinical practice [3]. Resection is only applicable to early patients. Although chemotherapy plays an important role in the treatment of colorectal cancer, it can only benefit some patients because of its large side effects and easy-to-produce drug resistance. Therefore, it is urgent to seek a new treatment scheme [4]. Traditional Chinese medicine is unique to China and has demonstrated amazing therapeutic effects. Whether it can be applied in the treatment of malignant tumors has become a research spotlight.

As a food-like tetrahydroxyflavone, kaempferol is found in various fruits and vegetables [5]. It possesses activities such as cardioprotection, neuroprotection, anti-inflammation, antidiabetes, antioxidation, and antitumor [6]. In this study, we explored the cancer inhibitory effect of kaempferol by evaluating the biological behavior of two human colon cancer cells intervened by this drug and investigating changes in the expression of MMP1, 2, and 9 genes.

During the development of malignant tumors, cells and extracellular matrix work together to create a malignant microenvironment suitable for the development of malignant tumors [7]. As a large family that takes metal ions (e.g., calcium and zinc) as cofactors, MMPs were discovered with the function of cleaving the extracellular matrix [8]. MMP1 is a collagenase that degrades the collagen of type I, II, and III, while MMP2 and MMP9 are gelatinases that degrade type-IV collagen [9]. The level of MMP1 expression is closely related to the prognosis of patients with colon cancer, and the mechanism of MMP1 may be associated with its involvement in the metastasis and spread of tumors. MMP2 is likewise involved in the adhesion between tumor cells and mesothelial cells, thereby triggering metastasis [10, 11]. Also, the expression of MMP9 is connected with the prognosis of colon cancer [12]. Therefore, MMP1, 2, and 9 genes act as a driving force in tumor invasion/metastasis and the formation of neovascularization within tumors.

## 2. Materials and Methods

### 2.1. Reagents

McCoy'S 5A medium (Lot No. 2020047) and calf serum (Lot No. 1829596) were purchased from Biological Industries, Israel. Trypsin-EDTA digestion solution (Lot No. 20200422) and penicillin-streptomycin solution (Cat. No. p1400) were purchased from Beijing Solarbio Science & Technology Co., Ltd., China. The TCM monomer composition: kaempferol (Cat. No. ALX-380-005-M010) was purchased from Cayman Chemical, USA. CCK-8 (Cat. No. K1018) was purchased from APExBIO Technology, USA. qRT-PCR : RNA extraction kit (Cat. No. DP430), reverse transcription amplification kit (Cat. No. KR118), and SuperReal fluorescence quantitative pre-mix reagent (SYBR Gree, Cat. No. FP205) were purchased from Tiangen Biotech (Beijing) Co., Ltd., China. PCR primers were synthesized by Sangon Biotech (Shanghai) Co., Ltd., China. Western blot: lysis buffer, SDS gel preparation reagent, SDS-PAGE, PVDF membrane, and ECL reagent were purchased from Beyotime Biotechnology, Shanghai, China. Rabbit anti-human MMP1 (Cat. No. YF-PA13182), MMP2 (Cat. No. YF-PA13183), and MMP9 (Cat. No. YF-MA14266) monoclonal antibodies (primary antibodies) were purchased from Affinity Biosciences, USA. Rabbit anti-human GAPDH monoclonal antibody (primary antibody, Cat. No. ET1601-4) was purchased from Hangzhou Huabio, China. Horseradish peroxidase (HRP)-labeled sheep anti-rabbit polyclonal antibody (Cat. No. S1002) was purchased from SeraCare Life Sciences, USA. Annexin V-EGFP/PI dual-stained cell apoptosis assay kit (Cat. No. CA004-1) was purchased from Nanjing Signalway Antibody, China. Matrigel gel (Cat. No. M8370) was purchased from Beijing Solaibao Technology Co., Ltd., China.

### 2.2. Bioinformatic Analysis

The TCM monomer composition and its associated target genes were analyzed and selected using the HERB database. Then, the structures of target proteins were predicted using the protein 3D structure database ALPHAFOLD (https://alphafold.ebi.ac.uk/). Meanwhile, the Integrative Pharmacology-based Research Platform of Traditional Chinese Medicine (TCMIP) (https://tcmspw.com/tcmsp.php) was utilized to predict the molecular structure of the monomer composition. Following that, molecular docking analysis was performed to detect the correlation and docking mode between the selected monomer composition and the target genes.

### 2.3. Cells and Cell Culture

Human colon cancer cell strains HCT-116 and HT29 were purchased from Shanghai Biomedical, China. McCoy'S5A culture medium containing 10% fetal bovine serum and 1% double antibodies (100 U/mL penicillin and 100 *μ*g/mL streptomycin) was loaded into a 25-cm^2^ flask which was then incubated in an incubator (37°C, 5% CO_2_, and 95% humidity). Cell morphology, differentiation level, and cell density were monitored using an optical inverted microscope. Cells were digested by trypsin at a concentration of 0.25% for subculture after the monolayer cell density reached 80%–90%. The cells used in this study were all in the logarithmic phase with good viability [13].

### 2.4. Cell Grouping and Drug Treatment Methods

Based on diverse treatment methods, the cells were separated into: the blank control group experimental cells + culture medium; the normal control group: PBS + experimental cells + culture medium; and the Kae group: kaempferol + experimental cells + culture medium. The powdered experimental drug was dissolved with dimethyl sulfoxide (DMSO) and diluted with a serum-free culture medium to set the appropriate drug concentration gradient (see Table 1).

### 2.5. CCK-8 Assay Was Adopted to Detect the Half-Maximal Inhibitory Concentration of Kaempferol on the Two Types of Cells

Trypsin-digested HCT116 and HT29 cells in the logarithmic phase were inoculated into a 96-well plate at a density of 2,000 cells per well of 100 *μ*l and incubated in an incubator (37°C, 5% CO_2_, and 95% humidity) for 24 h. Then, kaempferol of different concentrations was added for 12-hour, 24-hour, and 48-hour incubation with six replicate wells set for each concentration. After reaching the specified time, 10 *μ*l of CCK-8 solution was added to each well for another 3-hour incubation. Finally, the absorbance (OD) was determined at 45 nm using a Rayto RT-6100 ELISA plate reader. The obtained data were used to plot standard curves with GraphPad and the half-maximal inhibitory concentrations (IC_50_ values) of kaempferol versus HCT116 and HT29 cells were calculated, where cell survival rate (%) = [(As−Ab)/(Ac−Ab)] × 100. In this equation, “As” represents the absorbance of experimental wells (absorbance of wells containing cells, drug-containing medium, and CCK-8); “Ab” represents the absorbance of blank wells (absorbance of wells containing normal medium and CCK-8); and “Ac” represents the absorbance of control wells (absorbance of wells containing cells, normal medium, and CCK-8). Cell inhibition rate (%) = 1 − cell survival rate [14, 15].

### 2.6. Wound Healing Assay

HCT116 and HT29 cells in the logarithmic phase were used after trypsin digestion and inoculated into a 6-well plate at a density of 6 × 10^5^ cells per well of 2 ml. The cell fusion rate was close to 100% after 24 h of incubation. A 20-*μ*l pipette tip was used to make vertical scratches on the bottom of the 6-well plate (3 strips per well). Then, the plate was washed three times with PBS solution. Following that, a serum-freedrug-containing medium was added for continued incubation. Finally, images were collected at the appropriate time using a Nikon TS2-S-SM inverted microscope and then processed using ImageJ software (version 8.0, Madison, WI, USA) to calculate the proportion of cell-free area in the treated group versus the control groups [16, 17].

### 2.7. Transwell Cell Migration Assay

Cells in the logarithmic phase were selected for trypsin digestion and then collected and resuspended by adding a serum-free culture medium as well as a culture medium containing kaempferol. The cell concentration was adjusted to 5 × 104, and 100 *μ*l of cell suspension was loaded into the upper transwell chamber. 700 *μ*l of complete culture medium containing 15% fetal bovine serum was loaded into the lower chamber with care to avoid generating air bubbles. The transwell chamber was then placed in the incubator for continued incubation. 48 h later, the chamber was taken out of the incubator. The original culture medium was discarded, and the transwell chamber was washed with PBS and fixed with 4% paraformaldehyde for 30 min. Thereafter, the chamber was washed again with PBS and then immersed in 0.5% crystal violet for 20 min. Following washing with PBS, the upper-layer cells that did not migrate were wiped off with a damp cotton swab, and the chamber was then air-dried. Finally, an inverted microscope was used to observe cell migration [17].

### 2.8. Transwell Cell Invasion Assay

Matrigel gel was diluted at 1 : 8 and then melted at 4°C overnight. 50 *μ*l of Matrigel gel was added to the bottom of the upper transwell chamber. Then, the chamber was well shaken horizontally and incubated at room temperature for 4 h until solidification. Cells in the logarithmic phase were selected for trypsin digestion and then collected and resuspended by adding a serum-free culture medium and serum-freekaempferol-containing culture medium. Then, the solution was inoculated into the chamber containing Matrigel gel at a concentration of 5 × 104 cells per 100 *μ*l. 700 *μ*l of complete medium containing 15% fetal bovine serum was added into the lower chamber and incubated for 48 h. After reaching the predetermined time, the chamber was taken out from the incubator and immersed in the well containing 700 *μ*l of 4% paraformaldehyde for 30-min fixation at room temperature. Then, the chamber was taken out and immersed in the well containing 0.5% crystal violet at room temperature for staining. 20 min later, the chamber was taken out and washed with PBS. Finally, the chamber was taken out and the number of transmembrane cells was then observed and randomly counted in three fields of view per well with three replicate wells set for each group [18].

### 2.9. Cell Apoptosis Detection

Annexin V and PI dual staining was adopted to detect cell apoptosis. In particular, HCT116 and HT29 cells in the logarithmic phase were inoculated into a 6-well plate at a concentration of about 6 × 10^5^ cells per 2 ml and incubated in an incubator (37°C, 5% CO_2_, and 95% humidity) for 24 h following drug treatment. Then, cells were collected and centrifuged at 1,000 × *g* for 5 min. The supernatant was discarded; 1 ml of precooled PBS was added; the cells were resuspended and centrifuged again, and the supernatant was discarded (this step was repeated twice). Following that, the binding buffer was diluted with DI water at 1 : 3, and 250 *μ*l of the solution was used to resuspend the cells. The cell concentration was adjusted to 1 × 10^6^, and 100 *μ*l of the cell suspension was pipetted into a 5-ml flow tube. Then, 5 ml of Annexin V/FITC and 10 ml of PI solution were added. Following proper mixing, the tube was incubated away from light at room temperature for 15 min. Finally, flow cytometry was used for analysis [18].

### 2.10. Cell Cycle Assay

Trypsin-digested cells were collected, washed with PBS, and then fixed with 70% ethanol solution at 4°C for 45 min. Following fixation, the cells were washed again with PBS, and the supernatant was discarded. Cells were resuspended using RNase buffer and incubated away from light at room temperature for 15 min. Then, PI was added to form a final light pink mixture, which was analyzed using flow cytometry [19].

### 2.11. qRT-PCR

Extraction of total RNA: Treated cells were digested by trypsin and then collected and centrifuged. The supernatant was discarded, and 30 *μ*l of PBS was added to resuspend the cells. 120 *μ*l of Solution R1 was then added and mixed for 30 sec. 500 *μ*l of Solution R2 was added and mixed well. The solution was then transferred to the adsorbent column, and 500 *μ*l of RNA washing buffer was added twice for washing. Finally, 20 *μ*l of nuclease-free H_2_O was added and centrifuged to obtain total RNA. 1 *μ*l of total RNA was used to determine the purity and concentration of total RNA on a UV spectrophotometer. cDNA reverse transcription: 2 *μ*g of total RNA was mixed well with 4 *μ*l of 5 × Fast Plus RT Master mix and 1 *μ*l of 20 × Oligo dT (25) &Random primer. Then, RNase-free H_2_O was added to supplement (available here) the mixture to 20 *μ*l. The reverse transcription condition was: 50°C, 5 min ⟶ 95°C for 1 min. Real-time fluorescence quantitative PCR: 2 *μ*l of cDNA was added to the mixed solution containing 10 *μ*l of 2 × ZAPA3G SYBR Green qPCR Mix, 0.4 *μ*l of PCR Forward Primer, and 0.4 *μ*l of PCR Reverse Primer (MMP1, MMP2, MMP9 primer sequences are detailed in Table 2; GAPDH was used as the internal reference). Then, RNase-free H_2_O was added to supplement the mixture to 20 *μ*l. The reaction procedure was: 95°C for 5 min, 1 cycle ⟶ 95°C for 10 s ⟶ 60°C for 30 s (fluorescence collection), 40 cycles. Results were set to CT values of 15–35 and solubility curves with single peaks (peak width <7 cells, peak height >800). Results were analyzed using the ΔΔCt relative quantification method, and the different level was expressed as 2^−ΔΔCt^, where ΔΔCt = experimental group (Ct target gene − Ct housekeeping gene) − control group (Ct target gene − Ct housekeeping gene) [20].

### 2.12. Western Blot

Cells in good condition in the logarithmic phase were used for trypsin digestion. Then, 200 *μ*l of cell lysis buffer was added and mixed well on ice by pipetting with a pipette tip. The mixture was then centrifuged at 14,000 × *g* for 5 min and the supernatant was used to get the proteins of the treated cells. BCA ELISA plate reader was used to determine the protein concentration at 562 nm. Then, an equal amount of buffer was added to the mixture which was then heated at 100°C for 5 min to denature the proteins. 10% SDS-PAGE separating gel and 5% concentrated gel were prepared, mixed well, and filled into the gel plate without delay. The comb was inserted and set aside for 30 min at room temperature. Then, electrophoresis was performed for protein separation after the gel plate solidified. Following electrophoresis, the membrane was transferred to the PVDF membrane, blocked with 5% skim milk powder, and placed on a shaker at room temperature for 2 h. TBST was used to wash the membrane three times (10 min for each time). The primary antibody of the target proteins was then prepared according to the dilution ratio specified in the antibody instruction (1 : 10000 for GAPDH; 1 : 1000 for MMP1, 2, and 9, respectively) and incubated at 4°C for 2 h. Following that, the membrane was washed with TBST three times (10 min for each time). The secondary antibody (1 : 10000) was incubated at room temperature for 2 h, and the membrane was washed with TBST three times (10 min for each time). Finally, ECL chromogenic technique was adopted; quantitative analysis was performed using ImageJ image analysis software; the relative expression of each group of proteins was expressed as the mean gray value (IOD) of the target lane [21].

### 2.13. Statistical Analysis

Measurement data were expressed as (*x* ± *s*); paired *t*-tests were used for comparison between two groups; count data were expressed as percentages; chi-squared tests were adopted for comparison among multiple groups. ImageJ 8.0, GraphPad 8.0, and Adobe illustrator software were used for image generation, and the significance of all statistical comparisons was set to *P* < 0.05.

## 3. Results

### 3.1. Bioinformatic Analysis of the Association and Docking Mode of Kaempferol and Target Proteins

**Figure 1:**
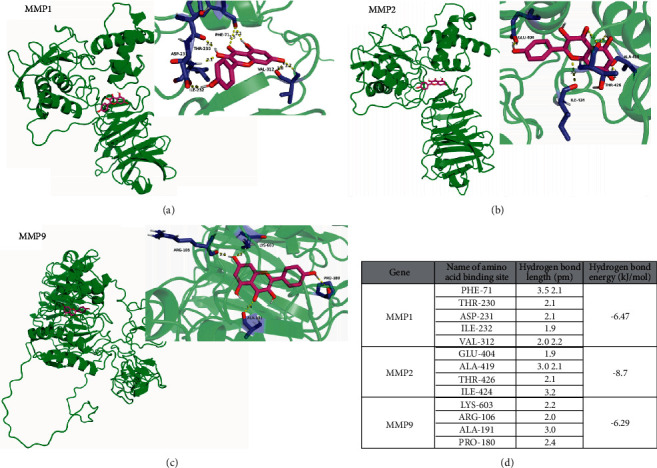
Bonding mode of kaempferol and amino acids of target proteins MMP1, 2, and 9 as well as hydrogen bond length and bond energy.

The TCM monomer composition (i.e., kaempferol) and target proteins MMP1 (ID : P03956), MMP2 (ID : P08253), and MMP9 (ID : P14780), which had a strong correlation with colon cancer, were screened using HERB database. Subsequent docking analysis revealed that in MMP1, kaempferol could be bonded to such amino acid sites as PHE-71, THR-230, ASP-231, ILE-232, and VAL-312 by hydrogen bonds, with a total bond energy of around −6.47 kJ/mol; in MMP2, kaempferol could be bonded to GLU-404, ALA-419, THR-426, and ILE-424, with a total bond energy of about −8.7 kJ/mol; in MMP9, kaempferol could be bonded to amino acid sites such as LYS-603, ARG-106, ALA-191, and PRO-180 by hydrogen bonds, with a total bond energy of about −6.29 kJ/mol (Figures 1(a)–1(d)).

### 3.2. Assessment of Viability of Two Types of Colon Cancer Cells Treated with Kaempferol

**Figure 2:**
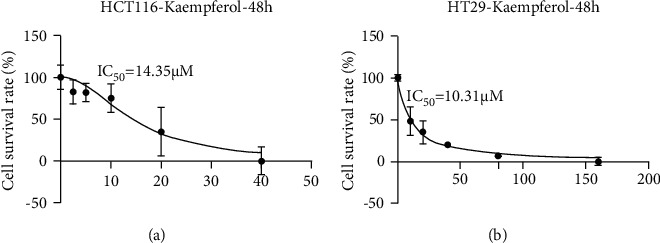
Changes in the survival rates of HCT116 and HT29 colon cancer cells. (a). shows that the IC_50_ value of HCT116 cells treated with kaempferol for 48 hours is 14.35 *μ*M. (b) shows that the IC_50_ value of HT29 cells treated with kaempferol is 10.31 *μ*M (*n* = 6).

CCK-8 assay was adopted to detect the inhibitory effect of kaempferol on the proliferation and viability of colon cancer cells. Standard curves were plotted with drug concentration as the horizontal coordinate and cell viability as the vertical coordinate. It was demonstrated that as drug concentration increased, the cell survival rate diminished (*P* < 0.0001). IC_50_ values were then calculated, and the 48th hour and its corresponding IC_50_ value were used as the intervention time and intervention concentration for the subsequent assays, respectively (Figures 2(a) and 2(b)).

### 3.3. Assessment of Cell Migration Ability after Drug Treatment Using Wound Healing Assay

**Figure 3:**
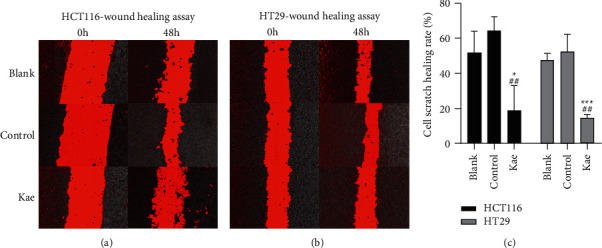
Assessment of cell migration ability. (a) and (b) show the migration ability of HCT116 and HT29 treated with kaempferol, while (c) shows the cell migration rate (*n* = 3); ^*∗∗∗*^*P* < 0.005, ^*∗*^*P* < 0.05 (blank control group vs. Kae group), ^##^*P* < 0.001 (normal control group vs. Kae group).

Photography was performed at 0 and 48 h following wound healing assay, and the scratch area was calculated using ImageJ software for comparison. Results suggested that the migration rate of HCT116 cells in the blank control group (51.60 ± 10.07% *n* = 3) and the normal control group (64.33 ± 6.54% *n* = 3) was far higher than that in the Kae group (18.55 ± 11.74% *n* = 3), with a statistically significant difference (*P* < 0.05). Likewise, the migration rate of HT29 cells was higher in the blank control group (47.44 ± 3.07% *n* = 3) and the normal control group (52.25 ± 8.07% *n* = 3) compared with the Kae group (17.40 ± 1.8% *n* = 3), with a statistically significant difference (*P* < 0.01) (Figures 3(a)–3(c)).

### 3.4. Assessment of Cell Migration Ability Using Transwell Migration Assay

**Figure 4:**
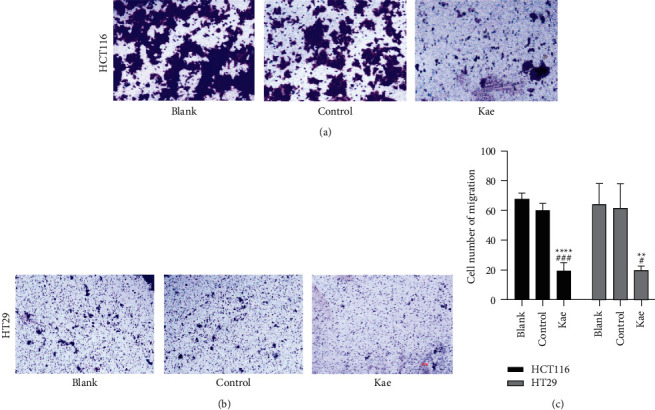
Assessment of cell migration ability. (a) and (b) show the cell migration ability of HCT116 and HT29 treated with kaempferol, while (c) shows the number of transmembrane cells (*n* = 3); ^*∗∗∗∗*^*P* < 0.0001, ^*∗∗*^*P* < 0.01 (blank control group vs. drug group), ^####^*P* < 0.0001, and ^#^*P* < 0.05 (normal control group vs. drug group). Scar bar means 100 px.

The numbers of HCT116 and HT29 transmembrane cells were calculated after 48 h with the following results. The number of HCT116 cells was higher in the blank control group (64.33 ± 11.14 *n* = 3) than that in the Kae group (20 ± 2.16 *n* = 3) with a statistically significant difference (*P* < 0.01); likewise, this number was higher in the normal control group (61.67 ± 13.27 *n* = 3) than that in the Kae group (*P* < 0.05), suggesting a statistically significant difference (*P* < 0.01). The number of HT29 cells was higher in the blank control group (68 ± 2.94 *n* = 3) than that in the Kae group (19 ± 4.55 *n* = 3) with a statistically significant difference (*P* < 0.0001); likewise, this number was higher in the normal control group (60 ± 3.74 *n* = 3) than that in the Kae group, indicating a statistically significant difference (*P* < 0.005). (Figures 4(a)–4(c)).

### 3.5. Transwell Invasion Assay Adopted to Detect the Invasion Ability of Colon Cancer Cells

**Figure 5:**
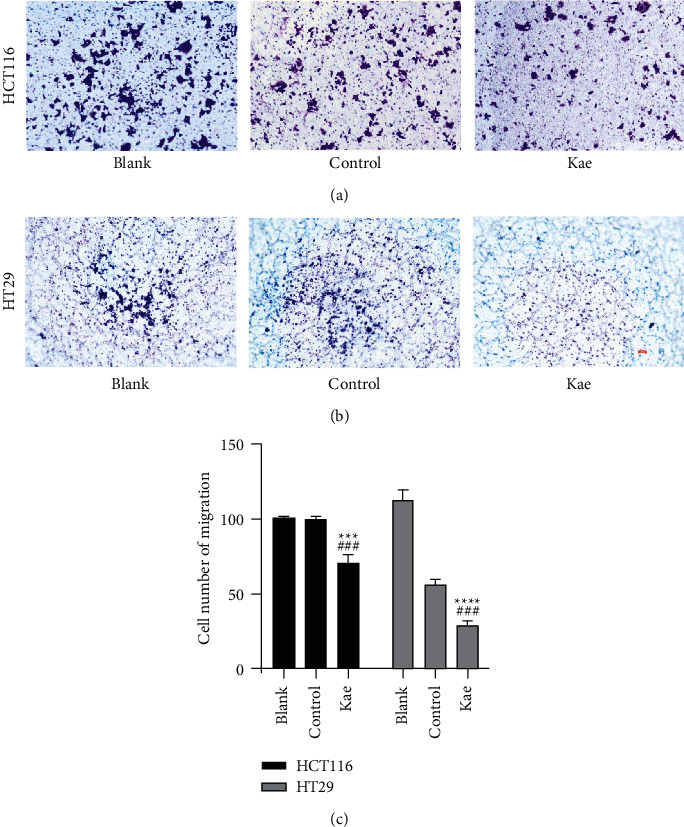
The invasion ability of colon cancer cells. (a) and (b) show the cell invasive ability of HCT116 and HT29 treated with kaempferol, while (c) shows the number of transmembrane cells. (*n* = 3); ^*∗∗∗∗*^*P* < 0.0001, ^*∗∗∗*^*P* < 0.005 (blank control group vs. drug group), and ^###^*P* < 0.005 (normal control group vs. drug group). Scar bar means 100 px.

Transwell invasion assay indicated that the number of transmembrane cells in the three groups of HT29 cells was as follows: 101 ± 0.82 *n* = 3 for the blank control group, 100 ± 1.63 *n* = 3 for the normal control group, and 71.33 ± 4.19 *n* = 3 for the Kae group. The number of HCT116 transmembrane cells in the blank control group was higher than that in the Kae group with a statistically significant difference (*P* < 0.0001); likewise, this number was higher in the normal control group than that in the Kae group (*P* < 0.005). The number of HCT116 transmembrane cells in the blank control group (112.33 ± 4.11 *n* = 3) was higher than that in the Kae group (29.33 ± 2.05 *n* = 3) with a statistically significant difference (*P* < 0.01); likewise, this number was higher in the normal control group (56.67 ± 2.49 *n* = 3) than that in the Kae group (*P* < 0.05) (Figure 5(a)–5(c)).

### 3.6. Flow Cytometry Adopted to Detect the Apoptosis of Tumor Cells after Drug Intervention

**Figure 6:**
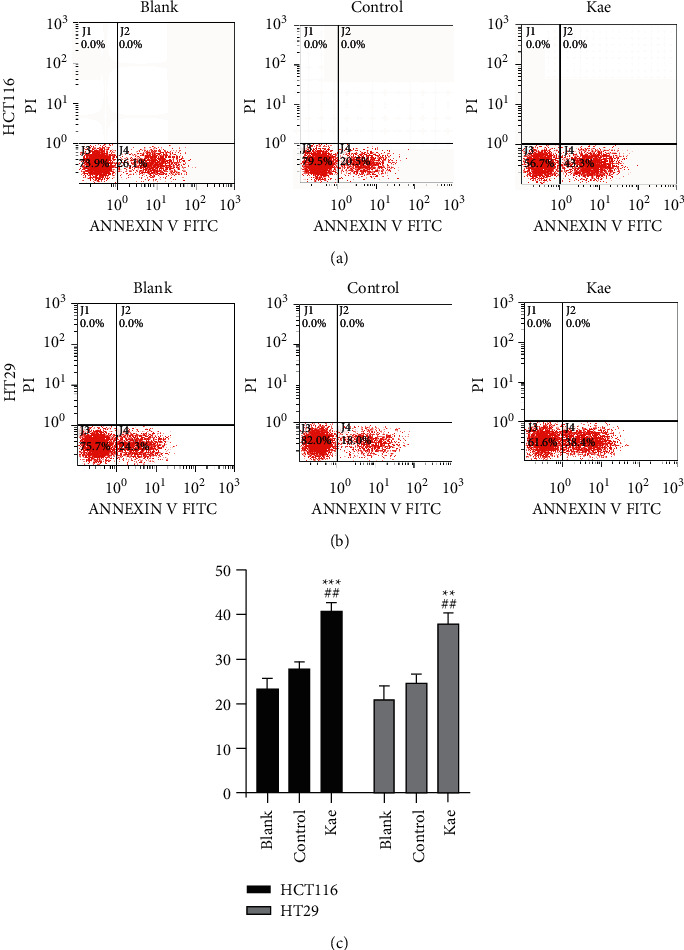
Kaempferol promotes apoptosis in tumor cells. (a) and (b) show the specific apoptosis of tumor HCT116 and HT29 cells, while (c) shows the relevant statistics. (*n* = 3); ^*∗∗*^*P* < 0.01, ^*∗∗∗*^*P* < 0.005 (blank control group vs. drug group), and ^##^*P* < 0.01 (normal control group vs. drug group). Scar bar means 100 px.

Compared to 23.2% *n* = 3 in the blank control group and 27.7% *n* = 3 in the normal control group, the early apoptosis rate of HCT116 cells in the Kae group was 40.8% *n* = 3 with a statistically significant difference (*P* < 0.01); this enabled kaempferol to facilitate the apoptosis of HCT116 cells. In HT29 cells, this rate in the blank control group (21.1% *n* = 3) and the normal control group (24.7% *n* = 3) was lower than that in the Kae group (37.9% *n* = 3), indicating a statistically significant difference (*P* < 0.01) (Figures 6(a)–6(c)).

### 3.7. Flow Cytometry Adopted to Detect Changes in the Cell Cycle of Tumor Cells after Drug Intervention

**Figure 7:**
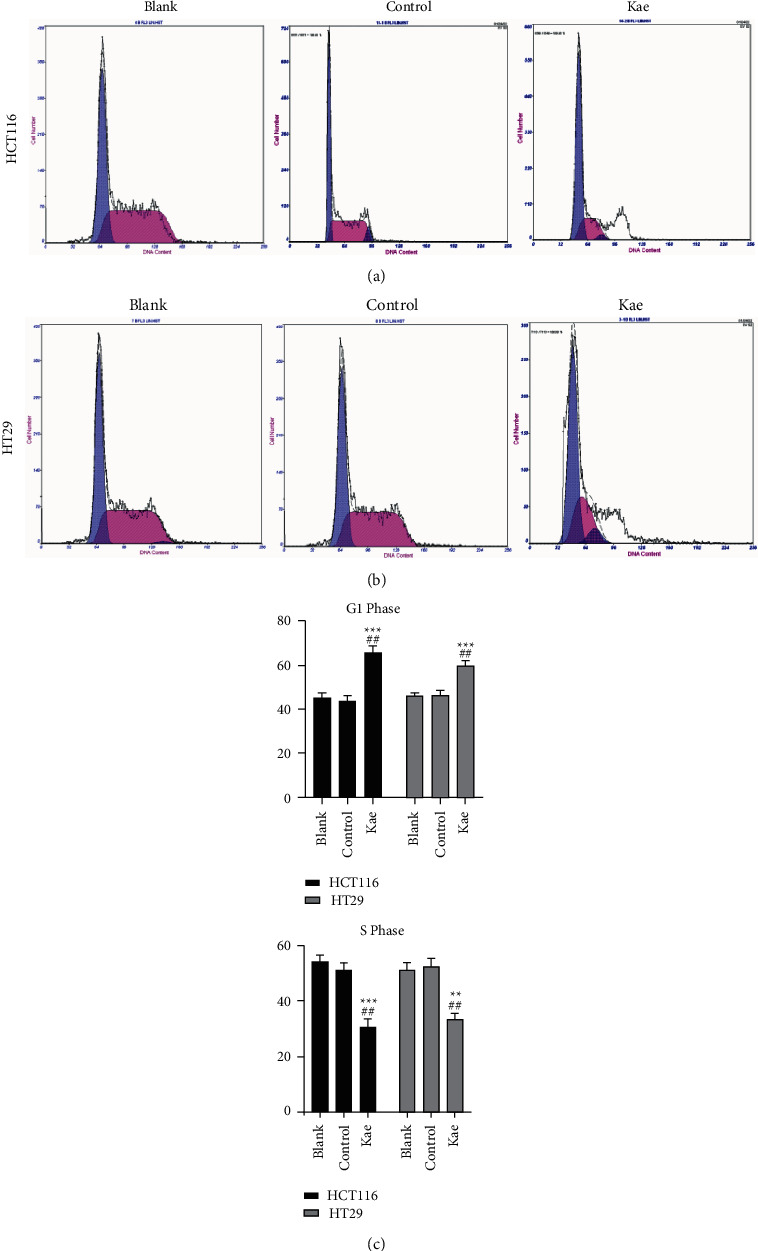
Effect of kaempferol on the cell cycle of colon cancer cells. (a) and (b) show the cell cycle changes in tumor HCT116 and HT29 cells, while (c) shows the relevant statistics. (*n* = 3); ^*∗∗∗*^*P* < 0.005, ^*∗∗*^*P* < 0.01 (blank control group vs. drug group), and ^##^*P* < 0.01 (normal control group vs. drug group).

Kaempferol was used to treat the two types of human colon cancer cells, namely HCT116 and HT29, and the following results were revealed. The proportion of tumor cells in the G1 phase was higher in the Kae group compared to the blank and normal control groups, indicating a statistically significant difference (*P* < 0.005 and *P* < 0.01, respectively). By contrast, this proportion in the S phase was higher in both the blank and normal control groups than that in the Kae group with a statistically significant difference (*P* < 0.01 for both) (Figures 7(a)–7(c)).

### 3.8. qRT-PCR Adopted to Detect the Expression of Target Genes in Tumor Cells after Drug Intervention

**Figure 8:**
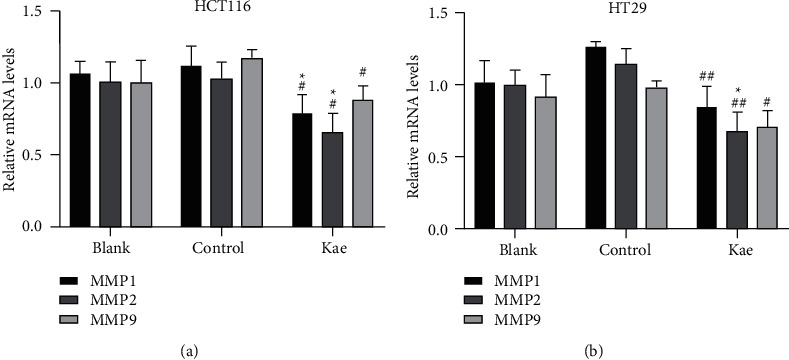
Expression of target gene. (a) and (b) show the effect of kaempferol on MMP1, 2, and 9 gene expression in colon cancer cells. (*n* = 3); ^*∗*^*P* < 0.05 (blank control group vs. drug group), ^#^*P* < 0.05, and ^##^*P* < 0.001 (normal control group vs. drug group).

In HCT116 cells, the expression of MMP1 (*t* = 3.068, *P*=0.0374, *n* = 3) and MMP2 (*t* = 3.221, *P*=0.0080, *n* = 3) in the blank control group was higher than that in the Kae group with a statistically significant difference, while no significant difference was observed in the expression of MMP9 (*t* = 1.182, *P*=0.3026, *n* = 3); the expression of MMP1 (*t* = 3.018, *P*=0.0393, *n* = 3), MMP2 (*t* = 3.836, *P*=0.0185, *n* = 3), and MMP9 (*t* = 4.442, *P*=0.0113, *n* = 3) was higher in the normal control group than that in the Kae group, indicating a statistically significant difference. In HT29, MMP2 (*t* = 3.461, *P*=0.0258, *n* = 3) expression was higher in the blank control group than that in the Kae group with a statistically significant difference, while no significant difference was observed in the expression of MMP1 (*t* = 1.415, *P*=0.2301, *n* = 3) and MMP9 (*t* = 2.058, *P*=0.1087, *n* = 3); the expression of MMP1 (*t* = 4.905, *P*=0.0080, *n* = 3), MMP2 (*t* = 4.887, *P*=0.0081, *n* = 3), and MMP9 (*t* = 4.239, *P*=0.0133, *n* = 3) was higher in the normal control group than that in the Kae group, suggesting a statistically significant difference (Figures 8(a) and 8(b)).

### 3.9. Western Blot Adopted to Detect the Expression of Target Proteins in Tumor Cells after Drug Intervention

**Figure 9:**
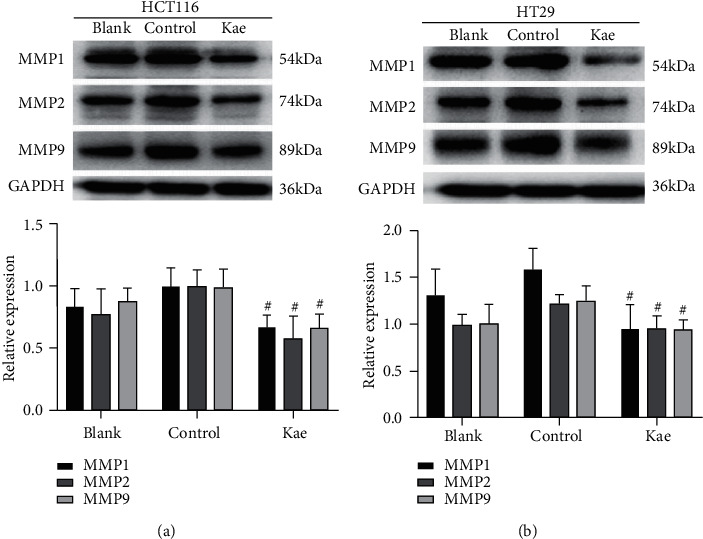
Expression of target protein. (a) and (b) show the effect of kaempferol on the intracellular expression of MMP1, 2, and 9 proteins in colon cancer cells. (*n* = 3); ^#^*P* < 0.05 (normal control group vs. drug group).

In HCT116, no significant difference was revealed in the expression of MMP1 (*t* = 1.545, *P*=0.1971), MMP2 (*t* = 1.228, *P*=0.2686, *n* = 3), and MMP9 (*t* = 2.331, *P*=0.0801, *n* = 3) between the blank control group and the Kae group; the expression of MMP1 (*t* = 3.061, *P*=0.0058, *n* = 3), MMP2 (*t* = 3.229, *P*=0.0320, *n* = 3), and MMP9 (*t* = 3.029, *P*=0.0388, *n* = 3) in the normal control group was higher than that in the Kae group with a statistically significant difference. Likewise, in HT29, no significant difference was observed in the expression of MMP1 (*t* = 1.597, *P*=0.1856, *n* = 3), MMP2 (*t* = 0.3032, *P*=0.7768, *n* = 3), and MMP9 (*t* = 0.4271, *P*=0.6913, *n* = 3) between the blank control group and the Kae group; by contrast, the expression of MMP1 (*t* = 3.115, *P*=0.0357, *n* = 3), MMP2 (*t* = 2.892, *P*=0.0445, *n* = 3), and MMP9 (*t* = 2.889, *P*=0.0446, *n* = 3) in the normal control group was higher than that in the Kae group with a statistically significant difference (Figures 9(a) and 9(b)).

## 4. Discussion

Natural compounds draw increasing interest because of their anticancer effects at the molecular level. Kaempferol is a kind of flavonoid, which widely exists in a variety of natural foods and drugs [22]. MMPs is a family of calcium, zinc, and other metal ion-dependent endopeptidases, of which MMP1, 2, and 9 are three important members [23]. MMP1 can cleave many components of the extracellular matrix and promote tumor proliferation. Its expression is closely related to tumor size and grade [24]. MMP2 plays a very important role in the occurrence and development of malignant tumors because of its degradable ECM components [23]. MMP2 promotes the lysis of fibronectin (FN) and vitronectin (VN), thereby enhancing the adhesion of protein hydrolytic fragments with adhesive properties; it triggers tumor metastasis due to its involvement in the adhesion of tumor cells to mesothelial cells [10, 11]. MMP9 displays substrate specificity for type-IV collagen and FN; it promotes epithelial-mesenchymal transition (EMT) by cleaving FN, thus enabling the release of transforming growth factor-*β* (TGF-*β*) and promoting tumor growth [24].

Epigenetics is an important component that controls the development and variation of single cells, such as DNA methylation, histone modification, and regulation of non-coding RNA [25]. Epigenetic modification plays an important role in the occurrence and development of colorectal cancer. Epigenetic changes can regulate transcription factors, inhibit the expression of tumor suppressor genes, and induce the overexpression of proto oncogenes, leading to the occurrence and development of cancer [26]. However, some natural derivatives inhibit the occurrence and development of malignant tumors by affecting epigenetic changes. In the study of Deb et al., it was found that epigallocatechin-3-gallate acid could increase H3K9/18 acetylation of matrix metalloproteinase-3 promoter region in human prostate cancer cells by downregulating EZH2 and histone H3K27, thereby reactivating TIMP3 gene expression [27]. Meanwhile, Mitsiogianni et al. found that sulforaphane can regulate the downstream proteins of histone deacetylase (HDAC) 1, 2, and 4 by changing the methylation and acetylation of lysine residues in melanoma cells to inhibit the development of tumor cells [28]. Kaempferol, as a monomer of traditional Chinese medicine, also has similar effects. It can restrict the expression of (HDAC), reduce DNA methyltransferase 3b, downregulate miRNA-21, and upregulate miRNA-340 to inhibit the development of colon malignant tumors [26].

Bioinformatic selection, analysis, and preliminary studies revealed that kaempferol has an obvious inhibitory effect on the proliferation, invasion, and migration of malignant tumors. For instance, kaempferol can promote the release of apoptosis mediator cytochrome-c (Cyt c) through internal and external pathways such as the mitochondrial membrane and by activating caspase-8, thereby promoting cytoskeleton contraction, intracytoplasmic vesicle generation, and DNA lysis to promote cellular apoptosis [29]. Kaempferol can enhance the sensitivity of pancreatic cancer cells to targeted drugs by inhibiting PI3K/AKT signaling pathway and epidermal growth factor receptor [30]. In addition, kaempferol can inhibit the occurrence and development of prostate cancer by inhibiting the expression of Ki67 [31]. Kaempferol and TGF-*β* receptor (ALK5) binding inhibits the phosphorylation and translocation of Smad2 and reduces the expression of Smad4, thus inhibiting the proliferation, migration, and invasion of tumor cells [32]. To date, chemotherapy is still an important treatment for patients with colorectal cancer. However, the huge side effects should not be underestimated. For example, fluorouracil causes nausea, vomiting, and other gastrointestinal reactions; and platinum anticancer drugs cause neurotoxicity. This series of adverse reactions led to the abandonment of treatment in some tumor patients, and finally delayed the opportunity of treatment. As a unique treatment method in China, traditional Chinese medicine has recorded the diagnosis and treatment of tumors as early as in the classic Chinese medicine Yellow Emperor's Inner Canon [33]. With the characteristics of small side effects, low price, easy access to nature, and significant curative effect, it has gradually come into the world's vision. Based on this characteristic of traditional Chinese medicine, this study started from the basic experiment to study the anticancer effect of kaempferol, a single traditional Chinese medicine component, on colorectal cancer, so as to lay a foundation for subsequent animal and clinical experiments and expand the road for the development of new anticancer drugs. According to previous studies, some have confirmed that kaempferol can affect the biological behavior of colon cancer cells, such as proliferation, invasion, and migration, but the specific mechanism is still not clear. Based on the above research, this study proposed the hypothesis of the biological effects of MMP1, 2, and 9 on colon cancer; jointly screened the monomer Chinese medicine component kaempferol through bioinformatics and traditional Chinese medicine database (https://www.herb.com); and jointly tested the previous hypothesis through HCT116 and HT29 colon cancer cells, thus strongly proving that MMP1, 2, and 9 can affect the occurrence and development of colon cancer cells.

In this study, the CCK-8 assay revealed that TCM monomer compositions displayed a significant inhibitory effect on the proliferation of colon cancer cells; the median inhibitory concentration for HCT116 was 14.35 *μ*M. This is slightly larger than the half inhibition concentration (IC50 = 3.326 *μ*M) previously studied by Budisan et al. [13]. Meanwhile, the IC_50_ value of kaempferol for HT29 is 10.31 *μ*M, which is far less than the IC_50_ value (50 *μ*M) obtained by Pham et al. [34]; the wound healing assay and transwell migration assay showed a significant decrease in cell migration after the intervention of the aforementioned monomer composition; likewise, transwell invasion assay suggested that the invasion ability of the cells following the above treatment was also decreased. In addition, flow cytometry revealed that kaempferol could block the cell cycle of colon cancer cells and promote the apoptosis of tumor cells. This result is consistent with the results of Han et al. and Sezer et al. [14, 35]. Moreover, the expression of the target genes MMP1, 2, and 9 was lower in the drug intervention group compared with the two control groups as detected by qRT-PCR; the expression of the three target proteins in the control groups and the Kae group was determined by western blot technique, with the result that the expression of the target proteins in the control groups was higher than that in the Kae group.

Nonetheless, it has its shortcomings. First, only fundamental experiments were conducted, with the absence of supportive evidence from animal and clinical trials. Second, only a single gene was investigated, and no further study was conducted on the signal pathways that affect the biological behavior of colon cancer. As a dietary flavone, kaempferol has the limitations of fast metabolism, low solubility, and low bioavailability, which has become a major obstacle to its anticancer effect. However, this study did not provide relevant research on improving its bioavailability [36].

Kaempferol, the TCM monomer composition used in this study, demonstrated a significant inhibitory effect on colon cancer cells. It blocked the cell cycle of tumor cells; inhibited the proliferation, invasion, and migration of tumor cells; and induced their apoptosis. Meanwhile, this drug reduced the expression of MMP1, 2, and 9 genes in colon cancer cells, which might be related to the decreased invasion and migration ability of tumor cells. This also confirmed the correlation between kaempferol and MMP 1, 2, and 9, which was consistent with the results of our previous bioinformatic analysis.

## Figures and Tables

**Table 1:** Concentration gradient of kaempferol acting on two human colon cancer cells.

TCM monomer composition	Cell strain	Drug concentration (*μ*M)
Kaempferol	HCT116	2.5.5.10.20.40
	HT29	10.20.40.80.160

**Table 2:** Primer sequences of target genes.

Primer name	Specific sequence	Amplicon size (bp)
MMP1	Forward 5′-ATGAAGCAGCCCAGATGTGGAG -3′	137
	Reverse 5′-TGGTCCACATCTGCTCTTGGCA -3′	

MMP2	Forward 5′-AGCGAGTGGATGCCGCCTTTAA -3′	138
	Reverse 5′-CATTCCAGGCATCTGCGATGAG -3′	

MMP9	Forward 5′-GCCACTACTGTGCCTTTGAGTC -3′	125
	Reverse 5′-CCCTCAGAGAATCGCCAGTACT -3′	

## Data Availability

The data used in this study are available at https://www.jianguoyun.com/p/DcwGwNAQ1OvBChj5scoEIAA.

## Supplementary Materials

Supplementary Materials Supplementary Figure 1. Graphic abstract. Description of the supplementary figure: the purpose of this figure is to illustrate that we analyzed that kaempferol and matrix metalloproteinases (MMP) 1, 2, and 9 can be stably connected by hydrogen bonds through bioinformatics and traditional Chinese medicine-related databases. Then, we selected two human colon cancer cell lines HCT116 and HT29 and intervened the two cells with kaempferol, respectively, by detecting the changes in cell proliferation, migration, invasion, cell cycle, and apoptosis. Then, the expression of MMP1, 2, and 9 in each group of cells was detected to prove the effect of kaempferol on the biological behavior of colon cancer cells and verify the results of previous biological analysis. The hypothesis that kaempferol inhibits the occurrence and development of colon cancer cells by affecting the expression of target genes was put forward.
