# Supplementary material for: Effect of Kaempferol on the Biological Behavior of Human Colon Cancer via Regulating MMP1, MMP2, and MMP9
Source: J Oncol. 2022 Sep 13;2022:2841762. doi: 10.1155/2022/2841762 (PMC9489381; doi:10.1155/2022/2841762)
Supplement: Supplementary Materials — Supplementary Figure 1. Graphic abstract. Description of the supplementary figure: the purpose of this figure is to illustrate that we analyzed that kaempferol and matrix metalloproteinases (MMP) 1, 2, and 9 can be stably connected by hydrogen bonds through bioinformatics and traditional Chinese medicine-related databases. Then, we selected two human colon cancer cell lines HCT116 and HT29 and intervened the two cells with kaempferol, respectively, by detecting the changes in cell proliferation, migration, invasion, cell cycle, and apoptosis. Then, the expression of MMP1, 2, and 9 in each group of cells was detected to prove the effect of kaempferol on the biological behavior of colon cancer cells and verify the results of previous biological analysis. The hypothesis that kaempferol inhibits the occurrence and development of colon cancer cells by affecting the expression of target genes was put forward. [file 2841762.f1.docx]

**Suppl Fig 1. Graphic abstract.**


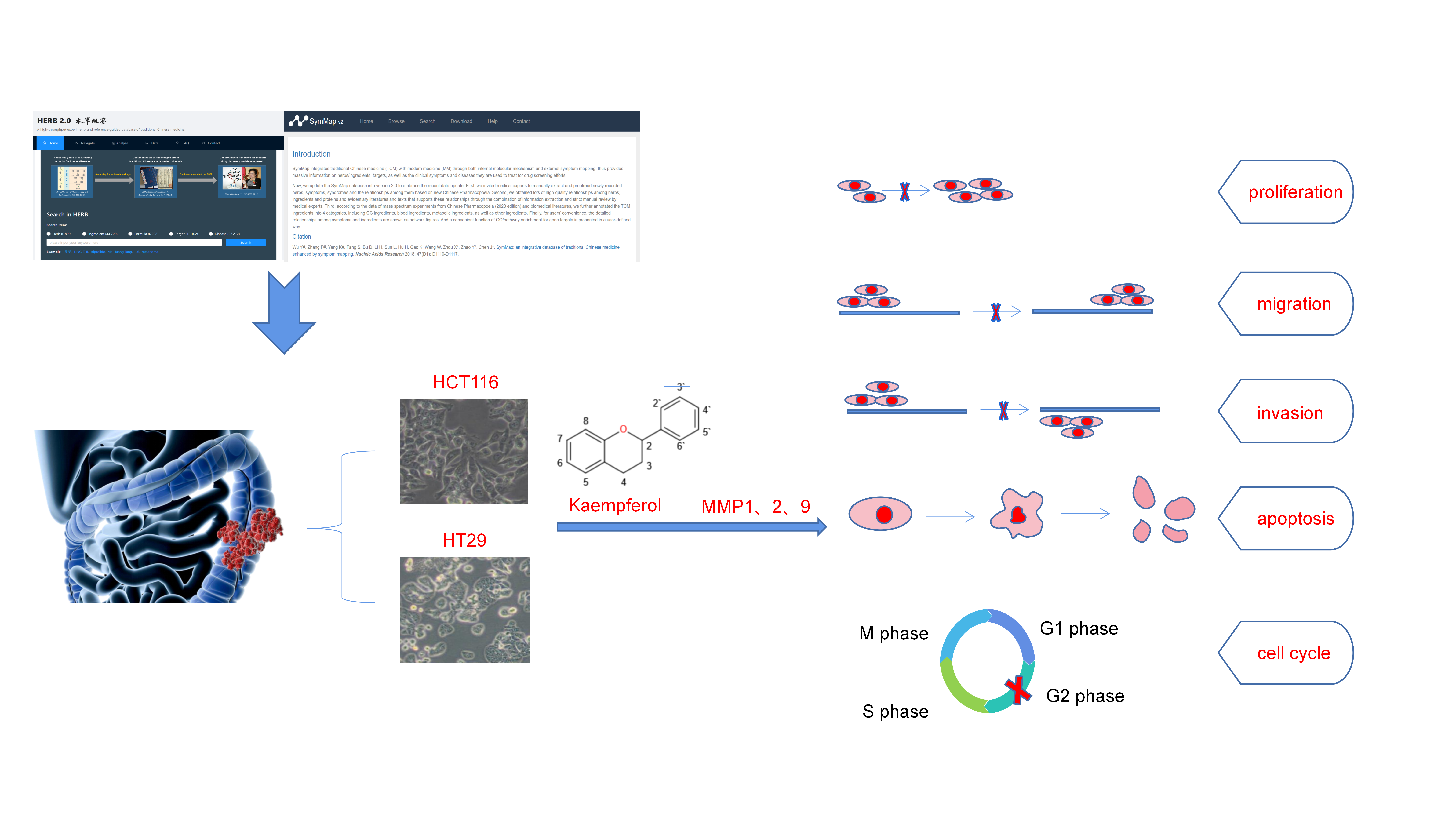


Description of supplementary Figure : The purpose of this figure is to illustrate that we analyzed that kaempferol and matrix metalloproteinases (MMP) 1, 2 and 9 can be stably connected by hydrogen bonds through bioinformatics and traditional Chinese medicine related databases. Then we selected two human colon cancer cell lines HCT116 and HT29, and intervened the two cells with kaempferol respectively, by detecting the changes of cell proliferation, migration, invasion, cell cycle and apoptosis, Then the expression of MMP1, 2 and 9 in each group of cells was detected to prove the effect of kaempferol on the biological behavior of colon cancer cells and verify the results of previous biological analysis. The hypothesis that kaempferol inhibits the occurrence and development of colon cancer cells by affecting the expression of target genes was put forward.
